# Neuroimaging Findings in Chronic Hepatitis C Virus Infection: Correlation with Neurocognitive and Neuropsychiatric Manifestations

**DOI:** 10.3390/ijms21072478

**Published:** 2020-04-02

**Authors:** Matteo Tagliapietra, Salvatore Monaco

**Affiliations:** Department of Neurosciences, Biomedicine and Movement Sciences, University of Verona, 37129 Verona, Italy

**Keywords:** hepatitis C, neurocognitive disorders, MR spectroscopy, diffusion tractography, functional MRI, PET-CT, SPECT

## Abstract

Chronic hepatitis C virus (HCV) infection is commonly associated with neurocognitive dysfunction, altered neuropsychological performance and neuropsychiatric symptoms. Quantifiable neuropsychological changes in sustained attention, working memory, executive function, verbal learning and recall are the hallmark of HCV-associated neurocognitive disorder (HCV-AND). This constellation is at variance with the neuropsychological complex that is seen in minimal hepatic encephalopathy, which is typified by an array of alterations in psychomotor speed, selective attention and visuo-constructive function. Noncognitive symptoms, including sleep disturbances, depression, anxiety and fatigue, which are less easily quantifiable, are frequently encountered and can dominate the clinical picture and the clinical course of patients with chronic HCV infection. More recently, an increased vulnerability to Parkinson’s disease among HCV-infected patients has also been reported. The degree to which neurocognitive and neuropsychiatric changes are due to HCV replication within brain tissues or HCV-triggered peripheral immune activation remain to be determined. Without absolute evidence that clearly exonerates or indicts HCV, our understanding of the so-called “HCV brain syndrome”, relies primarily on clinical and neuropsychological assessments, although other comorbidities and substance abuse may impact on neurocognitive function, thus confounding an appropriate recognition. In recent years, a number of functional and structural brain imaging studies have been of help in recognizing possible biological markers of HCV-AND, thus providing a rationale for guiding and justifying antiviral therapy in selected cases. Here, we review clinical, neuroradiological, and therapeutic responses to interferon-based and interferon-free regimens in HCV-related cognitive and neuropsychiatric disorder.

## 1. Introduction

Chronic hepatitis C virus (HCV) infection is associated with major hepatic and extrahepatic complications, including a number of neurological conditions [1,2].

Neuropsychiatric symptoms and neurocognitive dysfunction frequently occur in patients with chronic HCV infection, independent of liver disease severity or the rate of HCV replication. Sleep disturbances, depression and fatigue, in addition to reduced quality of life, are often associated with neurocognitive decline in subjects with non-cirrhotic chronic HCV infection, and may even dominate the disease course.

Neuropsychological tests have been used for cognitive assessment in HCV-associated neurocognitive disorder (HCV-AND) and include different batteries for global cognition, frontal-lobe function, mood, attention, memory and behavioral evaluation.

HCV-AND shows a profile of prevailing frontal lobe involvement characterized by disordered executive function, with impairment of working memory, processing speed, set-shifting, decision-making and verbal fluency [3,4,5,6], in association with apathy and disinhibition [7]. Dysfunction of the prefrontal cortex can also result in alexithymia, the inability to recognize and express one’s feelings, thus affecting patient-reported symptoms [8].

The attentional function necessitates the complementary action of both frontal and parietal cortices, with the former involved in the targeting of a particular stimulus (selective attention), and the latter in maintaining that focus (sustained attention). In HCV-AND, sustained attention seems to be the more commonly affected subdomain [3], whereas episodic memory per se is relatively less affected, possibly owing to attentive disturbances and defective encoding strategies [3,9]. An uncommon feature is the impairment of cognitive domains related to posterior brain regions that affect constructional praxis, visuo-spatial and visuo-perceptual tasks.

Comorbid conditions, as hepatic encephalopathy (HE) and human immunodeficiency virus (HIV) infection, could also alter mentation in HCV-infected patients. Minimal HE in particular refers to the paucisymptomatic cognitive effect of a liver disease of sorts, detectable only with tailored neuropsychological testing as a prevalent impairment of selective attention, visuo-spatial deficits with impaired navigation, increased reaction time with executive dysfunction and loss of inhibitory controls [10]. A multimodal approach is needed to discern the impact of HCV-AND and minimal HE on cognitive function, with assessment of liver function, instrumental evaluations and extended neuropsychological testing not exclusively focused on frontal executive function.

Intriguingly, there are also genetic factors that play a role in the development of the neurocognitive syndromes and neuropsychiatric symptoms in HCV-infected patients. Available data on the apolipoprotein E (APOE) genotype show that expression of the ε4 allele, an important risk factor for Alzheimer’s disease and with ε2 of cerebral amyloid angiopathy [11], results in milder impairment in attention and executive function in HCV-AND [12].

Here we review the spectrum of HCV-related neuroimaging changes associated with cognitive and neuropsychiatric changes which are subsumed under the terms HCV-AND and “HCV brain syndrome”, and we also examine the effects of antiviral therapy in subjects treated with old and new anti-HCV drugs.

The following sections are devoted to different imaging techniques employed in the study of HCV-AND (Table 1) and obtained results (Table 2).

## 2. Imaging in Hepatitis C Virus-Associated Neurocognitive Disorder (HCV-AND)

### 2.1. Nuclear Medicine

Regional hypometabolism on 18-fluorodeoxyglucose positron emission tomography (^18^FDG-PET) in HCV-AND has been observed in anterior cingulate, superior and middle frontal gyri, mainly correlating with impairment of attention and higher fatigue scores, in addition to the parahippocampal gyrus and bilateral caudate nuclei, changes associated with anxiety/depression and increased reaction time on attentive and free recall testing. A relative hypometabolism of part of the cerebellum is also reported [17], a finding incongruent with pathological studies excluding a direct cerebellar involvement, and as such, is suggestive of a remote effect of fronto-striatal involvement with reduced cerebellar innervation, as observed after distant cerebrovascular insults [49].

Diffuse serotoninergic projections from midbrain nuclei modulate the activity of cortical and subcortical structures, in particular of those involved in the regulation of behavior, mood, and attentive functions [50]. Conversely, motor activity and decision-making processes are tuned by both cognitive and emotional abilities, which are mediated by midbrain dopaminergic bundles that reverberate signals between the cortex and different levels in the basal ganglia [51].

Compared to healthy controls, decreased striatal dopamine transporter uptake (<2 standard deviations) has been documented in 60% of HCV-infected subjects, and this finding has been associated with decline in executive-attention tasks [20]. In addition, a positive correlation between striatal dopaminergic innervation and cerebral metabolism, as evaluated with ^18^FDG-PET, was also evident in most cortical areas and basal ganglia [17]. Serotoninergic innervation is similarly affected in 50% of these patients [20], although modulation of whole brain metabolism has been observed only in the presence of concomitant dopaminergic impairment [17]. Microglia activation, as assessed with ^11^C-(R)-PK-11195-PET, is observed in the frontal cortices of HCV-seropositive subjects only in the presence of peripheral clearance of the virus, and in the temporal cortex in those without concomitant fatigue [19]. In patients with intact cognition, caudate uptake has been observed, with thalamic uptake being restricted to selective HCV genotypes [18].

A protective role on cognition of the immune system activation is hinted by an inverse correlation of the inflammatory response in caudate, putamen and thalami with scores of attention function [19], although uptake in the same region is also observed to correlate with peripheral blood viral load [18].

Inconclusive data are available on the very early course of HCV infection, evaluated on HIV-infected cohorts, documenting a normal ^11^C-(R)-PK-11195-PET profile [47]. A confounding factor to be accounted for comes from the possibility of concomitant alterations due to HE. On ^11^C-(R)-PK-11195-PET, HE usually determines an increased uptake, especially in basal ganglia and the thalamus, that accrues along with cognitive deterioration [52].

### 2.2. Perfusion-Weighted Imaging (PWI)

Hypoperfusion due to reduced neuronal activity has been observed in the left frontal cortex and, to a lesser degree, in the right frontal cortex, the temporoparietal cortices and posterior cingulate gyrus in a group of naïve HCV-positive subjects with attention impairment. In this group, hyperperfusion of the basal ganglia suggests immune activation secondary to HCV invasion leading to a vascular response [24], a phenomenon observed also in other inflammatory conditions, such as multiple sclerosis [53].

### 2.3. Structural Magnetic Resonance Imaging (MRI)

Several cortical regions demonstrated substantial structural alterations. Left frontal and bilateral occipital lobe atrophy was noted on cortical thickness assessment, unrelated to the level of fatigue [26]. HCV also accrued the characteristic frontal atrophy of alcohol abuse [27].

On voxel-based morphometry analysis and magnetization transfer ratio, atrophy and cytoarchitectural alterations were observed in the insular lobe, a structure involved in goal-oriented attention, and in the adjacent parietal operculum. Atrophy was also noted in the amygdala and parahippocampal regions following serial assessments [9].

Alterations have been observed also in structures without proven viral invasion on pathological studies, suggesting local remodeling due to deafferentation. So far, secondary involvement has been observed both as atrophic changes, seen in the bilateral thalami, and as pseudohypertrophic changes, seen in several cerebellar grey matter regions [9].

White matter abnormalities on the other hand have been observed only in HIV/HCV coinfected subjects [54] but not in paucisymptomatic HCV-monoinfected patients [33].

### 2.4. Diffusion Tensor Imaging (DTI)

In HCV-infected subjects, reduced local complexity with increased anisotropy is observed in the basal ganglia, correlating mainly with semantic fluency, and in the bilateral thalami [4]. Thalamic efferents, as the superior thalamic radiation, also show structural alterations that correlate with impairment on tests of attention and executive functions [31].

In the normal appearing white matter, Thames et al. observed an altered diffusivity mostly affecting the external capsule, made up of corticocortical, corticostriatal and striatocortical fibers connecting the basal ganglia and frontal cortex, and the fronto-occipital fasciculus, involved in semantic language processes and goal-oriented behavior. Diffusion tensor imaging (DTI) alterations correlate with frontal white matter inflammatory profile on Magnetic Resonance Spectroscopy (MRS) and poorer overall performance on neuropsychological testing, in particular in verbal fluency [4]. A similar pattern of involvement of association tracts with reduced anisotropy in fronto-occipital fasciculus and anterior commissural fibers has been described also by Bladowska et al. [24] and by Prell et al. using voxel-based morphometry [9].

Altered DTI indices were also observed in HIV/HCV-coinfected patients in normal appearing white matter as a whole, with HCV-infected status resulting in widespread alterations of diffusivity and anisotropy [15]. In particular, reduced anisotropy was observed in frontal and temporal white matter, and increased diffusivity in temporal and occipitoparietal white matter [29]. HIV/HCV-coinfected patients also exhibit damage to anterior commissural fibers, with HCV infection accounting for a more severe involvement of those fibers connecting the primary motor cortices [30]. Lastly, a single study observed an alteration of middle cerebellar peduncles, possibly secondary to deafferentation [24].

### 2.5. Magnetic Resonance Spectroscopy (MRS)

Cortical gray matter displays a paucity of alterations on most instances [18,35,37,38,39,40,41,42] except for a few MRS studies observing reduced neuronal population as portrayed by lower N-acetyl-aspartate (NAA) in posterior regions with [43] or without [45] concomitant increases in choline concentration.

Frontal white matter frequently displays increased choline [35,37,38,43,44] or, less frequently, myo-inositol concentrations that correlate with impairment of executive functions [4,46]. A significant increase in inflammatory markers is a far less prevalent finding in the posterior white matter [40], although it still correlates with performances on verbal and visual memory testing [43]. Decreased neuronal density, on the other hand, is an uncommon observation in both anterior [24,35,36] and posterior regions [4,37], with the latter correlating with visual memory and visuo-spatial alterations [43].

Neuronal loss in the striatum has been documented in a single study [43] while increased choline has been commonly observed [18,24,40,43,44] as well as, less often, findings of myo-inositol elevation [18,47]. Other groups report negative results [4,35,41,42] possibly related to the use of different sampling methods, with positive results usually exploring a larger voxel placed between several deep structures. To date, there is an absence of multivoxel studies in HCV-AND to rule out a heterogenous involvement of these structures. Inconclusive data are available on the very early course of HCV infection evaluated on HIV-infected cohorts, documenting a normal MRS spectrum [39] or an isolated scarce increase in myo-inositol [47].

The significance of inflammation in the striatum is still debated: MRS studies suggest a noxious effect on the neuropsychological profile [44], in particular affecting attentive and executive functions [18,43,47]. A confounding factor to be accounted for comes from the possibility of concomitant alterations due to HE. On MRS, a decrease of choline ratio in basal ganglia that correlates with HE severity could be observed [55].

### 2.6. Functional MRI (fMRI)

An altered function of frontal cortices elicits a remodeling of whole brain networks. On resting state fMRI, the relative importance of a specific node or cluster of nodes is expressed as centrality, and an increase of this value is thought to occur as a compensatory response to deficiencies in other nodes [56]. In HCV-infected patients, involvement of anterior structures is suggested by the compensatory increase in the right parietal cluster centrality and stronger connectivity in several temporo-parieto-occipital regions. A deficient frontoparietal attention network is thus relieved by the posterior regions, hyperactivated to assist in memory and attentive tasks. A successful remodeling and its efficiency determine the final cognitive profile. Better results demonstrate a positive correlation with increased centrality in the parietal cluster, as observed in affected subjects only. fMRI remodeling despite normal gray matter volumes on automated voxel-based whole-brain morphometry suggests that functional impairment actually predates structural alterations [33]. In other cases, HCV infection determines more subtle changes: in HCV-infected subjects faced with an obliged choice, impulsive behavior led to a similar pattern of activation in fronto-parietal areas irrespective of the difficulty of the choice, whereas in healthy controls an increased activation is observed [32].

Secondary involvement of distant structures can also be detected on fMRI, as higher cerebellar segregation due to involvement of cerebello-thalamo-cortical circuits [57].

Systemic inflammation, rather than local, can also exert cognitive effects. Increased peripheral blood prostaglandin E_2_, an inflammatory biomarker, has been associated with altered connectivity between the anterior insula and putamen and, in turn, with sickness behavior and increased perceived stress [58].

## 3. Response to Treatment

### 3.1. Interferon α (IFN-α)

Until recently, IFN-α and pegylated IFN-α have been the only effective treatments for HCV, showing rates of sustained virologic response (SVR), i.e., undetectable serum HCV RNA at 24 weeks after treatment completion, as high as 50–80% when associated with ribavirin [59]. Neuropsychiatric disturbances are a frequent event during treatment with these agents, with an overall prevalence as high as 80%, including mood disturbances (depression, irritability), psychosis, suicide ideation and impairment of cognitive functions [60,61,62].

Regardless of SVR achievement, transient treatment-induced depression can be observed in up to half of the patients on medication. The disorder fades with treatment suspension, being persistent at 6 months from the end of the treatment in less than one patient in ten [63]. Risk of further recurrences of depressive episodes after the treatment course is higher in male patients and those aged above 60 years, more so in case of premorbid anxiety or depressive disorder [64].

Major depressive disorder equally affects HCV-monoinfected and HIV/HCV-coinfected patients although, unexpectedly, the latter group usually displays less depressive symptoms [65]. Notably, ^18^FDG-PET studies have shown that, in HCV-infected patients, IFN-α therapy is associated with decreased metabolic activity prevalent in prefrontal cortices, and the extent of the observed changes covaries with depression scores [21]. These findings are in keeping with results of EEG spectral analysis showing anteriorization of alpha waves and increase in parietal theta waves paralleling IFN-induced depressive symptoms [66]. IFN-α affects in multiple ways the neurotransmitter network involved in mood regulation, whose description is beyond the scope of this article (see [67], in particular modulating both serotonin and dopamine pathways. Using ^18^F-dopa-PET and an fMRI paradigm that studied the hedonic reward circuitry, after IFN-α administration Capuron et al. observed inhibited dopamine release and dampened activation in the ventral striatum that correlated with depressive symptoms and reduced motivation. In particular, the uptake profile on PET imaging suggested a functional damage in the absence of frank neuronal loss, as manifested by increased uptake and decreased turnover of ^18^F-dopa [22].

A protective effect of IFN-α in HCV-infected subjects against Parkinson’s disease, a disorder characterized by dopaminergic impairment, is apparent as a reduction in incident cases as early as 5 years from a completed treatment course observed in epidemiological studies [68]. Apart from an effect on alpha-synuclein deposition, a possibility is that the achievement of SVR results in a smothering of neuroinflammation thus hampering the neurodegenerative process. The serotonin pathway, on the other hand, seems not to be affected by IFN-α treatment. A PET study with labeled 3-amino-4-(2-dimethylaminomethylphenylsulfanyl)-benzonitrile (^11^C-DASB), a ligand of the selective serotonin transporter, failed to disclose any significant differences in presynaptic serotonin transporter availability or the presence of any correlation with depressive symptoms after 8 weeks of treatment [23].

IFN-α acutely and rapidly affects the brain circuitry, impairing global network efficiency on a resting state functional MRI connectivity study [34], and resulting in a significant impairment in executive and learning domains [62]. Aptly timed neurophysiological evaluation with quantitative EEG testing [66] and event-related potential p300 [66,69] could also be sensitive and inexpensive methods to quantify IFN-related cognitive impairment.

There is less certainty on the chronic effect of this therapy. Although a normalization in basal ganglia inflammatory biomarkers on MRS in patients achieving SVR has been observed in both those groups with increased [42,48] and with decreased baseline values [41], in studies evaluating clinical outcomes opposing results of improvements on follow-ups in episodic memory, attention and phonemic verbal fluency [63], and also progressive worsening after treatment completion [59,62], have been observed. A possible explanation of these divergent outcomes relies on the influence on the cognitive domains of achieving SVR, and HIV serological status [59,63].

### 3.2. Direct-Acting Antiviral Agents (DAA)

The treatment of HCV infection has dramatically changed after the availability of direct acting antivirals, with sofosbuvir being the first widely introduced drug.

IFN-free regimens are generally well tolerated and highly effective, with SVR exceeding 95% in patients with compensated liver disease that completed the course [70]. Simple dosing and an acceptable side effect profile result in a good compliance, with >85% patients completing the treatment [71]. Neuropsychiatric side effects are usually limited to insomnia, occurring in <10% patients treated with direct-acting antiviral agents (DAA). Severe or life-threatening neuropsychiatric symptoms are rare but could occur, mostly in patients with baseline psychiatric risks. However, the prevalence of neuropsychiatric symptoms still seems to be lower than that observed during IFN therapy [72].

DAA as a group seems to be effective in alleviating depressive symptoms even in the short-term, an effect that persists after treatment completion, and with a significantly better profile if compared to IFN-treated patients [73,74].

A significant improvement in fatigue and depressive symptoms has been observed also in other studies with different DAAs either alone or in combination with ribavirin [75]. These effects are also observed when adding simeprevir to an IFN-ribavirin regimen, which results in a shorter worsening of fatigue and depressive symptoms in treated patients, especially among those achieving SVR [76].

The US National Institute of Health’s Patient-Reported Outcomes Measurement Information System^®^ (PROMIS^®^) is a tool used to better standardize the collection of patient-reported information on health symptoms and health-related quality of life domains, and is freely available [77]. Using this tool, clinically significant improvements in fatigue, sleep disturbances and functional well-being with minor improvement in most other assessed health measures were observed after a full course of treatment with DAAs, with improvement being strongly dependent on the achievement of SVR [78].

A decrease in the reported cognitive complaints, lamented by 68% of the patients at baseline and decreasing to 36% at follow-up, is observed with IFN-free therapy in both HCV-monoinfected and HIV/HCV-coinfected patients. Intriguingly, a normalization in executive function and visual memory scores on neuropsychological testing was evident as early as 12 weeks from treatment [79]. There are few other studies that tried to quantify the effects of DAAs on cognitive domains. An improvement in executive functions and visuospatial ability at a 24-week follow-up visit was observed with paritraprevir/ombitasvir/ritonavir+dasabuvir in a small group of patients infected with HCV genotype 1. Parallel evaluation with structural MRI disclosed a small reduction in several frontal cortical volumes, including the subcallosal area, which is involved in memories with emotional and spatial connotations, in addition to transverse frontopolar gyri, and the anterior segment of the insula [28].

Bladowska et al. observed an improvement in visual memory, executive and visuospatial domains after a treatment course of paritraprevir/ombitasvir/ritonavir+dasabuvir with or without ribavirin. DTI and PWI studies in this group also revealed greater anisotropy after therapy in several association, commissural and projection tracts and lower diffusivity in several association and projection tracts that correlated with changes in the neuropsychological profile. Higher basal ganglia perfusion after therapy was also observed, but this finding did not correlate with other neuropsychiatric and DTI changes [25]. In a pilot study, improvement of working memory, visual and verbal memory and executive function after treatment with ledipasvir-sofosbuvir correlated with several inflammatory biomarkers in peripheral blood, such as tumor necrosis factor-α, IFN-γ and catechol-O-methyl-transferase [80].

Although usually well tolerated, particular groups as cirrhotic patients could show a transient mild clinical worsening with slower reaction times and an increase in extra-slow components on EEG after treatment with DAA, unrelated to minimal HE, but resolving at follow-up [81].

## 4. Summary and Perspectives

HCV-brain syndrome presents as a prominent derangement of attention and executive functions, associated with neuropsychiatric symptoms. In accordance with this presentation, imaging studies show a prevalent involvement of frontal cortico-striatal structures and their connections, systems that regulate the interactions between emotional and motivation regulation, executive and motor functions [82].

At variance with metabolic and hepatic encephalopathy, a direct involvement of the brain is observed as an inflammatory reaction in these structures. Uncertainty still remains over the clinical significance of this inflammation, with inconsistent findings of either a noxious or protective effect on cognition in different studies.

Consistent with current hypotheses of brain circuitry, an altered function is also observed in distant structures, related to the frontal cortico-striatal network, in the absence of inflammatory findings.

Scarce evidence is available on the reversibility of imaging alterations after treatment, and their potential use as a biomarker to consider treatment initiation.

HCV-AND is a common complication in HCV-infected subjects and is a leading determinant of quality of life, although it is often underrecognized due to its subtlety. As effective and well-tolerated treatments are currently available, imaging biomarkers could assist clinicians in the evaluation of the cognitive involvement in HCV.

## Figures and Tables

**Table 1:** Overview of advanced imaging techniques.

**MRS**
MRS uses the magnetic resonance signal of a specific atom, usually hydrogen, to evaluate the concentration of selected cerebral metabolites in a particular volume [13]. Both single voxel and multivoxel assessments can be obtained. ∙ NAA: a neuronal metabolite. A decreased NAA represents neuronal dysfunction or loss; ∙ Choline (Cho): as a metabolite expressed in glial and proliferating cells, it is used as a marker of cell membrane turn-over and, possibly, inflammation; ∙ Myoinositol (mI): produced only by glial cells, it increases with glial proliferation or with myelin breakdown; ∙ Creatine (Cr): the reference metabolite, as its concentrations are relatively stable between tissues and conditions. [14]
**DTI**
DTI evaluates the ordered flow of water molecules, its magnitude and directionality along a restricted path, as it occurs in intact white matter. Data analysis can be based both on region/voxel-of-interest or on reconstructed neural tracts. ∙ Fractional anisotropy (FA): the degree of diffusion asymmetry in the different axes. Lower anisotropy represents white matter damage. Regions of interlacing white matter bundles with different orientations, or asymmetric loss of fibers instead result in higher anisotropy due to reduced complexity; ∙ Mean diffusivity (MD): overall spatial restraint due to cellular structures. Injury to any cellular tissue results in higher diffusivity. [15]
**PWI**
Neuronal activity directly correlates with brain blood flow in the small capillaries. On PWI, lower local neuronal activity can thus be observed as lower tissue perfusion, and quantified as cerebral blood volume (CBV) and cerebral blood flow (CBF).
**fMRI**
fMRI studies the oscillations of cortical activity as a function of subtle alterations in perfusion. The degree of concordance of these oscillations in between diverse regions suggests a functional coupling, due either to a direct or indirect connection between two regions [16]. Both resting state connectivity maps and evaluation of the activation pattern evoked by a certain task can be obtained.
**Structural MRI**
Structure labelling, parcellation and volume quantitation can be obtained with common MRI sequences using appropriate analytical systems.
**^18^FDG-PET**
^18^FDG-PET uses the metabolic signature of brain structures to investigate neuronal activity.
**^123^I-beta-CIT SPECT**
Dopaminergic and serotoninergic pathways can be assessed with ^123^I-beta-CIT to quantify DAT striatal uptake and SERT mesencephalic/hypothalamic availability.
**^11^C-(R)-PK-11195 PET**
PK-11195 binding is observed in activated microglia, infiltrating macrophages and astrocytes, supporting its use as an inflammatory marker.

NAA: N-acetyl-aspartate; MRS: magnetic resonance spectroscopy; DTI: diffusion tensor imaging; PWI: perfusion-weighted imaging; fMRI: functional magnetic resonance imaging; PET: positron emission tomography; SPECT: single photon emission computed tomography DAT: Dopamine Active Transporter; SERT Serotonin Transporter.

**Table 2:** Summary of the imaging findings in HCV-AND.

Nuclear Medicine	Response to Treatment
^18^*FDG-PET*—Cortical anterior > posterior and cerebellar hypometabolism [17] ^11^*C-(R)-PK-11195-PET*—Basal ganglia and thalamic microglial activation [18,19] ^123^*I-CIT-SPECT*—Decreased dopaminergic and serotoninergic innervation [17,20]	*IFN-α* Decreased anterior metabolism on ^18^FDG-PET [21] Inhibited dopamine release in ventral striatum [22] No effect on serotoninergic innervation [23]
**PWI**	
Cortical anterior > posterior reduced perfusion [24] Increased basal ganglia perfusion [24]	*DAA* Increased basal ganglia perfusion [25]
**Structural imaging**	
Reduced cortical thickness in frontal areas with [26] or without [27] occipital involvement Posterior insula and thalamic atrophy [9] Progressive amygdala and parahippocampal atrophy [9]	*DAA* Reduced frontal cortical volumes [28]
**DTI**	
Increased MD and reduced FA in global WM [15] more evident in fronto-temporal areas [29] and body of CC [9,24,30] Increased MD in external capsule [4] and fronto-occipital fasciculus [4,9,24] Decreased FA in bilateral middle cerebellar peduncles [24] and superior thalamic radiation [31] Increased FA in the basal ganglia [4]	*DAA* Increased FA in association, commissural and projection tracts [25] Decreased MD in association and projection tract [25]
**fMRI**	
Altered cortical connectivity possibly due to frontal deficit [32,33]	*IFN-α* Reduced ventral striatum activation [22] and global network efficiency [34]
**MRS**	
Decreased NAA in anterior [35,36,37] and posterior WM [4,37] Normal [18,35,37,38,39,40,41,42] or decreased NAA in cortical GM [43] Increased Cho in frontal [35,37,38,43,44] and posterior WM [40], cortical GM [45] and basal ganglia [18,24,40,43,44] Decreased [41] or normal Cho in basal ganglia [4,42] Increased mI in frontal WM [4,46], basal ganglia [18,47], cortical GM [45] and temporal structures [43]	*IFN-α* Basal ganglia inflammatory markers: decrease in groups with increased [48] and normal [42] baseline values increase in the group with decreased baseline values [41]

IFN-α: Interferon α; DAA: direct-acting antiviral agents; MD: mean diffusivity; FA: fractional anisotropy; WM: white matter; CC: corpus callosum; GM: grey matter; Cho: choline; mI: myoinositol.

## Abbreviations

HCV HCV-AND	Hepatitis C virus HCV-associated neurocognitive disorder
HE HIV	Hepatic encephalopathy Human immunodeficiency virus
^18^FDG PET	18-fluorodeoxyglucose Positron Emission Tomography
PWI	Perfusion-weighted Imaging
DTI	Diffusion Tensor Imaging
MRS	Magnetic Resonance Spectroscopy
NAA	N-Acetyl-Aspartate
fMRI	Functional Magnetic Resonance Imaging
IFN-α	Interferon α
SVR	Sustained Virological Response
^11^C-DASB	3-amino-4-(2-dimethylaminomethylphenylsulfanyl)-benzonitrile
DAA	Direct-acting Antiviral Agents
